# Ferrocene-Containing DNA Monolayers: Influence of Electrostatics on the Electron Transfer Dynamics

**DOI:** 10.1021/acs.langmuir.0c03485

**Published:** 2021-03-11

**Authors:** Ivan Magriñá, Mayreli Ortiz, Anna Simonova, Michal Hocek, Ciara K. O’ Sullivan, Robert J. Forster

**Affiliations:** † Departament d’Enginyeria Química, Universitat Rovira i Virgili, 26 Països Catalans, 43007 Tarragona, Spain; ‡ Institute of Organic Chemistry and Biochemistry of the Czech Academy of Sciences, Flemingovo nám. 2, CZ-16610 Prague, Czech Republic; § Department of Organic Chemistry, Faculty of Science, Charles University in Prague, Hlavova 8, CZ-12843 Prague 2, Czech Republic; ∥ Institució Catalana de Recerca i Estudis Avançats, Passeig Lluís Companys, 23, 08010 Barcelona, Spain; ⊥ School of Chemical Sciences, FutureNeuro SFI Research Centre, National Centre for Sensor Research, Dublin City University, Dublin D09 V209, Ireland

## Abstract

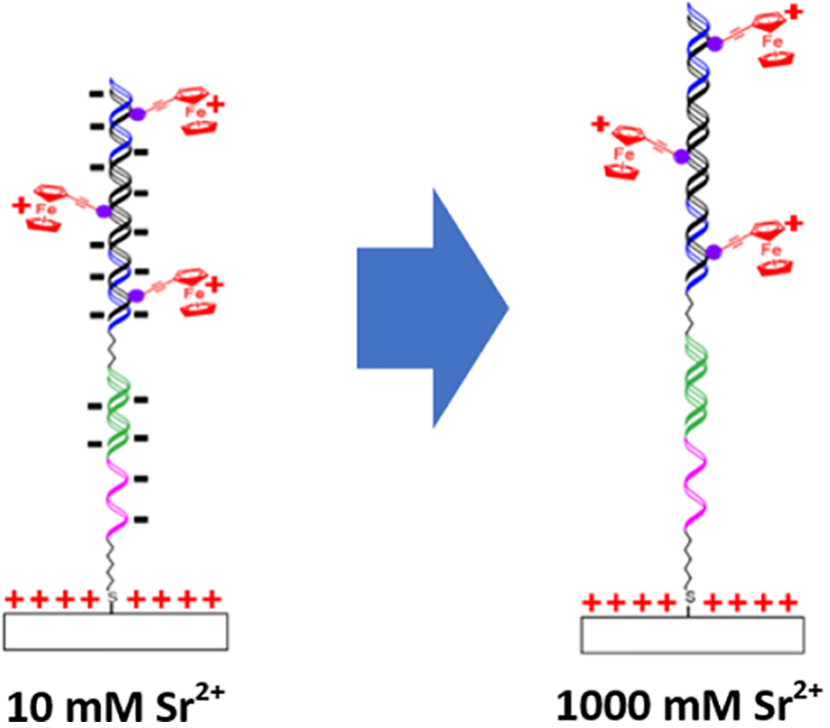

A 153-mer target DNA was amplified using ethynyl ferrocene dATP and a tailed forward primer resulting in a duplex with a single-stranded DNA tail for hybridization to a surface-tethered probe. A thiolated probe containing the sequence complementary to the tail as well as a 15 polythimine vertical spacer with a (CH_2_)_6_ spacer was immobilized on the surface of a gold electrode and hybridized to the ferrocene-modified complementary strand. Potential step chronoamperometry and cyclic voltammetry were used to probe the potential of zero charge, PZC, and the rate of heterogeneous electron transfer between the electrode and the immobilized ferrocene moieties. Chronoamperometry gives three, well-resolved exponential current–time decays corresponding to ferrocene centers located within 13 Å (4 bases) along the duplex. Significantly, the apparent standard heterogeneous electron transfer rate constant, *k*_app_^o^, observed depends on the initial potential, i.e., the rate of electron transfer at zero driving force is not the same for oxidation and reduction of the ferrocene labels. Moreover, the presence of ions, such as Sr^2+^, that strongly ion pair with the negatively charged DNA backbone modulates the electron transfer rate significantly. Specifically, *k*_app_^o^ = 246 ± 23.5 and 14 ± 1.2 s^–1^ for reduction and oxidation, respectively, where the Sr^2+^ concentration is 10 mM, but the corresponding values in 1 M Sr^2+^ are 8 ± 0.8 and 150 ± 12 s^–1^. While other factors may be involved, these results are consistent with a model in which a low Sr^2+^ concentration and an initial potential that is negative of the PZC lead to electrostatic repulsion of the negatively charged DNA backbone and the negatively charged electrode. This leads to the DNA adopting an extended configuration (concertina open), resulting in a slow rate of heterogeneous electron transfer. In contrast, for ferrocene reduction, the initial potential is positive of PZC and the negatively charged DNA is electrostatically attracted to the electrode (concertina closed), giving a shorter electron transfer distance and a higher rate of heterogeneous electron transfer. When the Sr^2+^ concentration is high, the charge on the DNA backbone is compensated by the electrolyte and the charge on the electrode dominates the electron transfer dynamics and the opposite potential dependence is observed. These results open up the possibility of electromechanical switching using DNA superstructures.

## Introduction

The structure of DNA can be exquisitely controlled to give “origami” structures with useful properties.^1^ Beyond the primary and secondary structures controlled by the nucleotide sequence and base-pairing of complementary nucleotides, respectively, DNA can respond in a reversible way to external stimuli such as pH, ions, temperature, and small molecules. These effects can be exploited to create nanomachine-like devices^2^ including pH-driven DNA motors,^3^ surfaces with reversible wettability,^4^ switchable DNA nanocompartments,^5^ and pH-responsive nanochannels able to discriminate between ions.^6^ Recently, it has been reported that Cu^+^ can be used to form an i-motif at neutral or slightly acidic pH with the process being reversed by chelating the Cu^+^ using ethylenediaminetetraacetic acid (EDTA) or by oxidizing it to Cu^2+^. This is the first example of redox-sensitive control of the DNA structure, which can be exploited to develop oxygen-sensitive nanomachines.^7^ Another metal that induces the formation of i-motifs at a neutral pH is Ag^+^.^8^ Interestingly, it is possible to construct a bipedal walker and stepper using two different motifs that respond to two different stimuli: (a) an i-motif structure sensitive to pH and (b) a thymine complex that responds to the presence of Hg^2+^.^9^ DNA can also respond to the presence of multivalent cations, such as Ca^2+^, Mg^2+^, cobalt(III)hexamine, or protonated forms of polyamines, i.e., putrescine, spermidine, or spermine.^10^ For example, a recent study showed that Ni^2+^ could control the separation between nanoparticles when dsDNA is used as a linker, decreasing the separation by up to 80%.^11^

In addition to these electrostatic interactions driven by ions in solution, the DNA structure can be influenced by applying an appropriate potential or electric field.^12^ Interfacial supramolecular assemblies of DNA on electrode surfaces represent an outstanding platform to investigate these effects. For example, Barton and co-workers demonstrated that it is possible to switch the orientation of tethered DNA duplexes from being essentially coplanar with the electrode surface to a perpendicular orientation using the applied potential to control the interaction of the negatively charged phosphate backbone with the electrode.^12^

Despite these advances, there have been a relatively few investigations of the combined effects of changing electrostatic interactions through the concentration, the identity of the ions in solution, and the potential applied to an interfacial DNA monolayer. For example, it may be possible to control the nature of the movement, e.g., to drive vertical, top–down, displacement. A significant advantage of this approach is that desirable structural changes could be driven reversibly at short timescales.

**Figure 1:**
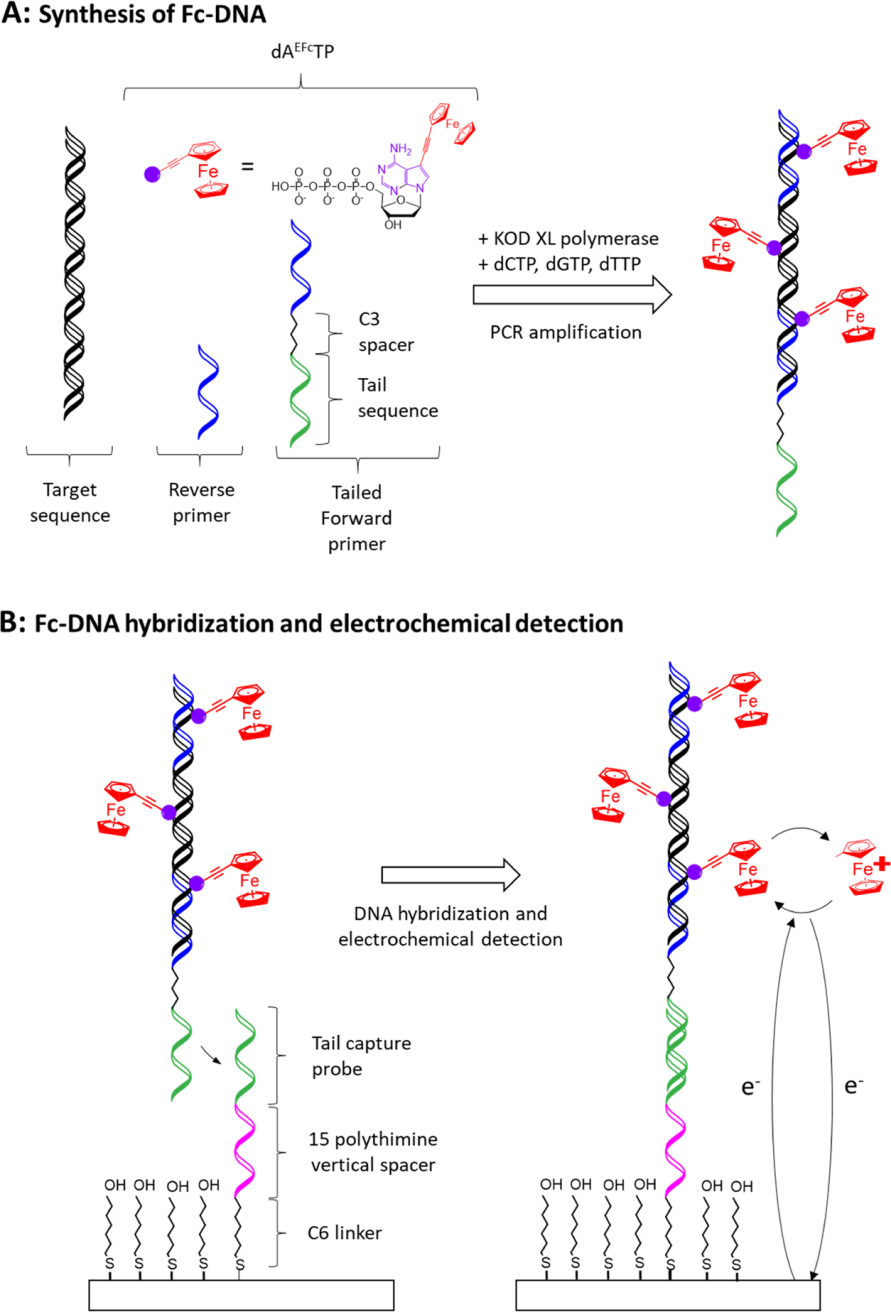
(A) Labeling of target DNA with ferrocenylethynyl by PCR in the presence of 7-(ferrocenylethynyl)-7-deaza-2′-deoxyadenosine triphosphate (dA^EFc^TP) and tailed forward primer. (B) Hybridization and electrochemical detection of the labeled DNA amplicon.

In this study, as illustrated in Figure 1, we have used the polymerase chain reaction (PCR) to produce a DNA duplex that is labeled with ferrocene groups attached at position 7 of 7-deazaadenine at different sites along the DNA duplex, which was immobilized on a gold electrode surface to probe electrostatic effects. The impact of DNA strand lateral separation has also been investigated. The divalent ion, Sr^2+^, has been used to modulate the electrostatic properties of the DNA by changing its concentration. Then, potentials that are either positive or negative of the potential of zero charge are applied. Once the DNA structure has equilibrated with the specific electrostatic conditions, the potential is stepped and the rate of electron transfer to the attached ferrocene groups is measured at various driving forces. Significantly, electron transfer occurs on a millisecond timescale and depends markedly on the potential applied, which is expected to influence the electrostatic interactions between the DNA and the electrode.

## Materials and Methods

### Reagents and Materials

KOD XL polymerase was purchased from Merck Millipore (Madrid, Spain), synthetic oligonucleotides were obtained from Biomers (Ulm, Germany) (Table 1), natural dNTPs were purchased from Thermo Fisher Scientific (Barcelona, Spain), and DNA free water was provided by Fisher Bioreagents. dA^EFc^TP was synthesized and characterized following the Sonogashira reaction, as previously described.^13^

**Table 1:** List of Oligonucleotide Sequences and Modifications^a^

oligo^bU^	sequence
*Karlodinium armiger* forward primer with tail (FwP-T)	5′-att acg acg aac tca atg aa −C3– ata gct tca cag cag agg tta caa c-3′
*Karlodinium armiger* reverse primer (RevP)	5′-aca cac atc caa cca tYt cac tg-3′
*Karlodinium armiger* target (KA)	5′-a**t**a gc**tt**ca cag cag agg **tt**a caa c**a**c c**aat**gc **t**gc **t**cc gc**ta**cc cgc g**at** c**t**c **at**g c**a**c c**a**g gg**a** gcg gc**aa**g**aa**gc c**a**g **a**gc **tt**c **aa**g **a**c**a** ccc c**ta** ccc ccg **t**gc **a**gg **a**gc **t**c**a** c**aaa**g**aaa**g **tt**c **a**c**a** gtg **a**g**a** tgg ttg g**a**t gtg tgt-3′
*Karlodinium armiger* thiolated capture probe (CP)	5′- ttc att gag ttc gtc gta att ttt ttt ttt ttt tt-3′-C6-SH

a Y represents the wobble C + T.

bU nderlined: sequences that correspond or are complementary to the primer sequences. In bold: nucleotides in the duplex that MIGHT incorporate a ferrocenylethynyl label. Important: Only **a** is modified with the ferrocenylethynyl label, **t** indicates that the **a** in the complementary strand has the label. There are at maximum 53-(ferrocenylethynyl)-7-deaza-2′-deoxyadenosine in total per dsDNA.

Electrode arrays were fabricated using soda-lime glass slides from Sigma-Aldrich (Spain), 3 mm thick poly(methylmethacrylate) (PMMA) from La Indústria de la Goma (Tarragona, Spain), and a double-adhesive gasket ARSeal 90880 from Adhesive Research (Ireland).

All other reagents were obtained from Sigma-Aldrich S.A. (Barcelona, Spain) and used as received. High-purity deionized water (18 MΩ cm^–1^) produced with a Milli-Q RG system (Millipore Ibérica, Spain) was used throughout. As a model system, amplification and detection of the toxic dinoflagellate *K. armiger* was used.

### Polymerase Chain Reaction Conditions

DNA amplicons were produced by the PCR in a T100 thermal cycler (Biorad) using the following protocol: 40 cycles at 95 °C for 30 s, 60 °C for 30 s, and 72 °C for 45 s, with a final elongation step at 72 °C for 5 min. The reaction mixture had 0.1 unit of KOD XL; KOD XL buffer 1×; 200 nM of each primer (Karlo FwP-T and Karlo RvP); dGTP, dCTP, and dTTP at 200 μM; dATP at 140 μM; **dA**^**EFc**^**TP** (60 μM); and KA target (1 nM).

### Electrode Functionalization with Capture Probe

Gold electrode arrays were fabricated on a soda-lime glass substrate, as previously described.^14^ The array consisted of three circular working electrodes and one rectangular reference electrode with surface areas of 1 and 4 mm^2^, respectively. To functionalize the working electrodes, the electrode array was first cleaned using Milli-Q water and a commercial soap (Vajillas Super, from Sosmi S.A. Spain), rinsed with Milli-Q water, and then dried with N_2_. A solution of the thiolated capture probe (1 μL) was placed on each working electrode and incubated overnight at 22 °C in a humidity-saturated chamber. The capture probe solution contained 1 μM capture probe (KA CP), 100 μM mercaptohexanol, and 1 M KH_2_PO_4_. Following functionalization, the array was rinsed with Milli-Q and dried with N_2_.

### Microfluidic Fabrication and Mounting

**Figure 2:**
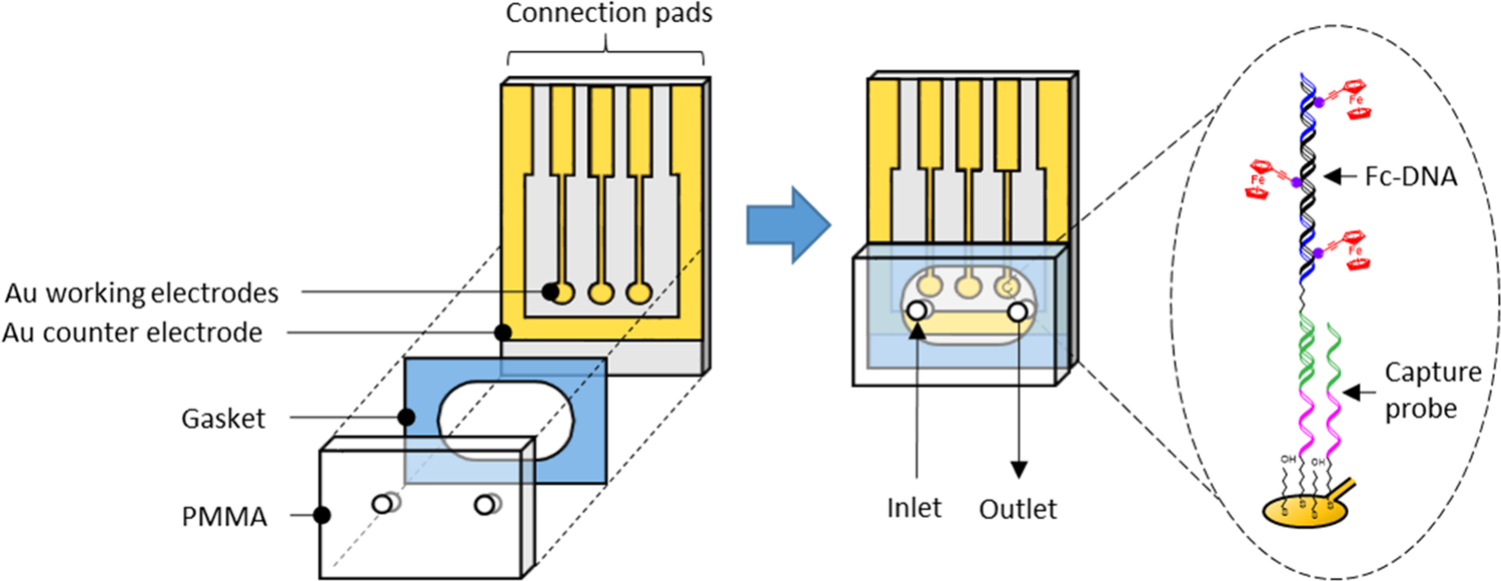
Schematic of electrode array showing the configuration and modification of the working electrode.

As illustrated in Figure 2, a 7 μL microfluidic chamber was constructed using a PMMA cover sealed to the electrode array using a double-adhesive gasket, thus defining the areas of the working and counter electrodes. To complete the electrochemical cell, an external Ag/AgCl reference electrode was placed in the droplet formed on top of the PMMA when 100 μL of the electrolyte solution was added to the microfluidic chamber.

### Amplicon Hybridization on Electrode Arrays

Following PCR, 7 μL of the PCR product was injected into the microfluidic chamber and hybridization took place at 22 °C in a humidity-saturated chamber for 30 min. The microfluidic chamber was then flushed three times with 200 μL of phosphate-buffered saline (PBS) Tween-20 and 200 μL of PBS. Prior to electrochemical measurements, the PBS solution was replaced with 200 μL of Sr(NO_3_)_2_ solution.

### Electrochemical Measurements

For cyclic voltammetry (CV) experiments, a PBSTAT 12 Autolab potentiostat/galvanostat and Nova 2.1.3 software were used. A preconditioning potential of 0 V was applied for 5 s and then cyclic staircase voltammetry was applied from 0 to 0.600 V using a 10 mV step at scan rates between 100 and 500–1000 mV s^–1^.

Short-time-scale chronoamperometry experiments were performed in a 5 cm^3^, single-compartment electrochemical cell that contained the DNA-ferrocene-modified electrode. Large-area Pt foil and a reference electrode were combined to form a counter electrode. The foil lowered the resistance and provided a high-frequency path. The current-to-voltage converter used a 1500 Ω feedback resistance and a response time of less than 10 ns. A custom-built programmable function generator-potentiostat was used. This instrument had a rise time of less than 10 ns and was used to apply potential steps of variable pulse width and amplitude directly to a two-electrode cell. The chronoamperograms were recorded using an HP54201A digital oscilloscope in a 64× time-average mode. Cell time constants were extracted by analyzing the short-time portion of the chronoamperograms using semilog current vs time plots.

## Results and Discussion

### Electron Transfer Mechanism and Double-Layer Structure

This system comprises different layers as shown in Figure 1B, i.e., the electrode surface, a mixed monolayer formed using mercaptohexanol and the thiolated C6 part of the capture probe, a flexible 15-mer ssDNA poly(T) vertical spacer, a stiff 19bp dsDNA capture sequence, a short but flexible C3 spacer, and finally a stiff 154bp dsDNA amplicon that contains a maximum of 54 ferrocenylethynyl redox labels distributed evenly along the dsDNA. Heterogeneous electron transfer to the ferrocene centers could occur via a DNA-mediated charge transport process or direct, through-space, electron transfer.

DNA-mediated charge transport is considered to be the dominant mechanism for redox probes intercalated within DNA duplexes and tethered to electrode surfaces.^15,16^ This mechanism is very sensitive to differences in the energy levels of adjacent bases, or mismatches in the duplex, e.g., a single bp mismatch can dramatically decrease the rate of electron transfer.^16^ The impact of coupling the redox labels to the DNA using short unsaturated linkers has been investigated,^17,18^ but intercalation of the redox probe may also occur in systems of this type.^19^

Long-range electron transfer can also occur by a through-space or direct electron transfer mechanism. For example, Plaxco et al.^20^ demonstrated this mechanism for ssDNA labeled with a terminal methylene blue tag and Anne et al. further demonstrated the mechanism for dsDNA, via elastic bending of the DNA duplex toward the electrode surface^21^ or by rotation of the dsDNA around the C6-anchoring linker.^22^

For the system considered here, the C3 linker and the 15T vertical spacer break the π-stacking, making it unlikely that long-range, DNA-mediated electron transfer is the dominant mechanism. Rather, through-space electron transfer that will be influenced by the motion of the dsDNA sequence containing the ferrocenes toward the electrode surface, e.g., driven by electrostatic interactions, is the expected electron transfer mechanism.

The double-layer structure is influenced by the electrode material,^23^ the relative surface coverage of the DNA and mercaptohexanol spacers, the state of charge of the DNA itself, and the composition of the supporting electrolyte. For example, Rant et al. demonstrated that DNA monolayers can be reversibly modulated or “switched” by periodically switching the applied potential.^24^ First, they concluded that a low DNA surface coverage is needed to minimize electrostatic repulsions within the DNA monolayer to allow the free rotational mobility of the strands.^25^ Applying a potential positive or negative from the PZC induces a redistribution of the dissolved ions within the double layer. Depending on the electrolyte concentration, strong electric gradient can exist at the interface that decays rapidly within a few nanometers depending on the electrolyte concentration,^23^ leading to significantly different field strengths across the length of the DNA strands.

### Cyclic Voltammetry

Cyclic voltammetry can provide deep insights into the local microenvironment of the ferrocene moieties through the formal potentials as well as the dynamics of electron transfer across the electrode/monolayer, as described by the standard heterogeneous electron transfer rate constant, *k*^o′^. Here, a 153bp PCR amplicon containing ferrocene-labeled deoxyadenosine and a single-stranded DNA tail was hybridized to an electrode-immobilized probe, which was complementary to the ssDNA tail. The ferrocene centers are located at discrete sites throughout the duplex, giving rise to different electron transfer distances that affects their individual electron transfer rates. Depending on the DNA surface coverage and the ferrocene loading, there may also be stabilizing or destabilizing lateral interactions as revealed by the peak widths in cyclic voltammetry. These effects are likely to be influenced by the electrolyte concentration and the cation identity since the cations can associate with the negatively charged DNA, thus changing the electrostatic charge on the DNA monolayer. In particular, multivalent cations can significantly change the secondary structure of the DNA, e.g., in solution, and for long dsDNA chains (e.g., λ bacteriophage genome), they can convert linear strands into a tightly packed, circumferentially wound torus.^26^ Also, B-DNA crystallographic structures for shorter sequences have shown that divalent cations are preferentially located in the major groove of the dsDNA and are able to modulate the structure of B-DNA and bend the helix toward the major groove.^27−29^ Theoretical models predict that multivalent cations are able to produce smooth (over 6bp) and large bending (20–40°) of these B-DNA.^10^ However, the effect of divalent cations on B-DNA structure in solution requires further investigation. Mirkin and co-workers suggest that divalent cations are able to alter the DNA structure on the molecular scale, enabling a reversible modulation of the “DNA length”,^11^ while Tajmir-Riahi and co-workers concluded that it is less evident that divalent cations are able to produce DNA conformational changes in the liquid phase.^30^ In close-packed monolayers, as well as these intramolecular interactions, the close proximity of the adsorbates can lead to interactions between adjacent strands.

**Figure 3:**
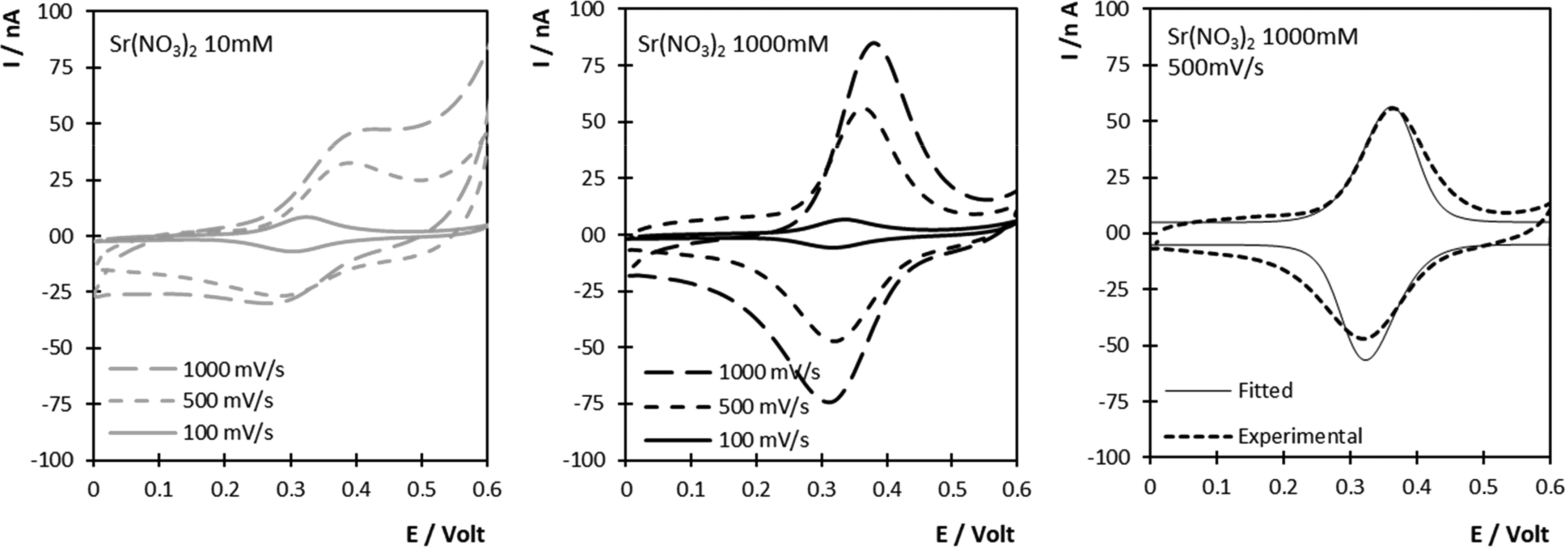
Cyclic voltammograms of target DNA hybridized on the electrode where the supporting electrolyte is 10 and 1000 mM Sr(NO_3_)_2_. The scan rates are 100, 500, and 1000 mV s^–1^, and an example of the best-fit voltammogram (500 mV s^–1^) obtained using a *k*_app_^o′^ value of 17.2 s^–1^ is shown.

Figure 3 shows the voltammetric responses obtained for the DNA duplexes using Sr(NO_3_)_2_ as the supporting electrolyte. In a previous publication, we observed that supporting electrolytes with divalent cations such as Sr(NO_3_)_2_, Ca(NO_3_)_2_, and Mg(NO_3_)_2_ allowed electron transfer from ferrocenes tethered to a dsDNA construct (similar to the one presented in this paper) to the electrode surface.^31^ In the present work, we chose Sr(NO_3_)_2_ as the supporting electrolyte because the peak current obtained was ≈10 to 20% higher than the peak currents obtained for Ca(NO_3_)_2_ and Mg(NO_3_)_2_. The concentrations of Sr(NO_3_)_2_ tested here were 10 and 1000 mM, and the scan rates studied were 100, 500, and 1000 mV s^–1^. For both electrolyte concentrations, well-defined peaks are observed corresponding to the oxidation and re-reduction of the bound ferrocenes. However, the peaks are significantly better defined in the more concentrated electrolyte and more ideally reflect the Gaussian response expected for surface-confined electroactive species. The DNA surface coverage, Γ, can be determined from the charge passed, *Q*, according to the equation Γ = *Q*/*nFA*, where *n* is the number of electrons transferred and *A* is the microscopic area of the electrode. Assuming that all ferrocene centers are electroactive at a slow scan rate in the presence of 1000 mM Sr(NO_3_)_2_, the charge passed under the background-corrected CV gives a surface coverage of 3.5 × 10^12^ ferrocenes·cm^–2^ or 5.8 × 10^–12^ mol cm^–2^. Taking into account that each dsDNA has a maximum of 53 ferrocenes (see Table 1), there is a low dsDNA surface coverage of 6.5 × 10^10^ molecules of dsDNA·cm^–2^, which is a prerequisite to obtain a switchable DNA layer (less than 10 × 10^11^ molecules/cm^2^ for a 48bp dsDNA).^33^ Indeed, it is possible to control the density of capture probes on the electrode surface by controlling the ratio of the lateral spacer (mercaptohexanol) and the capture probe.^32^ Nevertheless, the real dsDNA surface coverage in the presented system should be higher because the amplicon was produced by PCR using 30% dA^EFc^TP, not a 100% dA^EFc^TP. Due to the higher than calculated surface coverage and the fact that our DNA construct has an ssDNA15polyT vertical spacer, a 20-mer dsDNA capture sequence, aC3 spacer, and a 153bp dsDNA duplex, lateral interactions between neighboring hybridized DNA molecules might be expected. The peak shape and full width at half-maximum (FWHM) of the peak height can give insights into these lateral interactions. One electron reversible reactions involving surface-confined reactants show an FWHM of 90.6 mV. For the Fc-DNA systems, the FWHM values were 100–110 and 110–160 mV for the oxidation and reduction peaks, respectively, for both electrolyte concentrations used. FWHM values higher than 90.6/*n* mV (where *n* = number of electrons transferred) may suggest weak (<6.7 kJ mol^–1^) repulsive electrostatic lateral interactions between neighboring DNA strands. Another reason for the larger FWHM values could be different formal potentials for the individual ferrocene centers distributed along the DNA strand due to different local microenvironments.

The formal potentials at 10 and 1000 mM are 321 ± 1 and 288 ± 7 mV, i.e., there is a shift of 33 ± 7 mV in a negative potential direction and oxidation is thermodynamically more facile in the more concentrated electrolyte. For a strongly ion paired system, or where Donnan potential effects are dominant,^34^ one would expect a 59 mV per decade change in the electrolyte concentration. Here, the formal potential is relatively insensitive (33 ± 7 mV compared to the 118 mV predicted for ferrocenium–nitrate ion pairing) to the concentration of the supporting electrolyte, suggesting that charge-compensating ions are rather freely available within the monolayer and that the ferrocenium cation does not ion-pair significantly with the charge-compensating counterion, nitrate. Moreover, taking into account the surface coverage of the ferrocene centers, even at an electrolyte concentration of 10 mM, sufficient charge-compensating ions could diffuse within less than 0.5 ms, i.e., approximately 2000 times faster than the highest scan rate employed here. Thus, it appears that neither the availability nor the mass transport of charge-compensating counterions controls the rate of redox switching in this system. Rather, electron transfer to/from the remote ferrocenium/ferrocene centers appears to control the redox switching rate.

As reported previously,^35,36^ the dynamics of heterogeneous electron transfer to/from the ferrocenium/ferrocene couple is relatively insensitive to the identity of the supporting electrolyte, e.g., *k*^o′^ is approximately 555 ± 92 s^–1^ in 0.1 M HClO_4_, NaClO_4_, NaBF_4_, NaNO_3_, NaCl, and Na_2_SO_4_. Moreover, we find that the *k*^o^ value for ferrocene-methanol is 1.8 ± 0.3 cm s^–1^ where the Sr(NO_3_)_2_ concentration is varied from 0.1 to 1.0 M. It is perhaps important to note that *k*^o′^ depends weakly on the solvent, e.g., values of 0.042 ± 0.015, 0.048 ± 0.015, and 0.008 ± 0.002 cm s^–1^ have been reported in 0.1 M NaClO_4_/CH_3_CN, 0.1 M TBAClO_4_/CH_3_CN, and 0.1 M TBAClO_4_/CH_2_Cl_2_, respectively.^37^ Overall, these results suggest that while large changes in the local microenvironment around the ferrocene centers bound to the DNA could alter *k*^o′^, the dominant effect is likely to be from changes in the conformation of the DNA duplex rather than the intrinsic properties of the metal complex itself.

At a sufficiently slow scan rate, the voltammetric response of an ideal surface-confined redox-active species exhibits gaussian-shaped peaks, a peak-to-peak separation of zero, an anodic-to-cathodic peak current ratio (*i*_pa_/*i*_pc_) of unity, and a full width at half-maximum of 90.6/*n* mV, where *n* is the number of electrons transferred.^38^ When the time constant for cyclic voltammetry becomes comparable to that of heterogeneous electron transfer, the peak shape changes and a non-zero Δ*E* is observed. The small currents, <100 nA, ensure that the *iR* drop in these experiments is negligible even at the highest scan rate used, i.e., the relatively slow heterogeneous electron transfer causes the increased Δ*E*_p_. The redox centers in this system are located at different distances from the electrode surface, which means that the apparent standard heterogeneous electron transfer rate constant, *k*_app_^o′^, determined using CV will represent an average value across the entire population. However, voltammetry can provide clear insights into electrolyte dependent shifts in potentials as well as an indication of any changes in electron transfer rate.

The Laviron equation, which analyses the shift in the peak potentials as a function of the scan rate, can be used to determine *k*_app_^o′^. However, this approach does not allow deviations between the experimental and theoretical peak shapes, or scan-rate-dependent changes in the charge passed (fraction of ferrocenes that are electroactive at a given scan rate) or populations that contribute more significantly at different timescales, to be identified. Thus, *k*_app_^o′^ was determined by fitting the full experimental CV data to a surface-confined model in which the only adjustable parameter is the rate of heterogeneous electron transfer.^39^ The formal potential *E*^o′^, and surface coverage were determined using slow scan rate (1–5 mV s^–1^) voltammetry, where the rate of heterogeneous electron transfer rate does not influence the behavior. The rate constant was systematically varied using a gradient search method to minimize the summed square of the residuals between the experimental and theoretical currents. As shown in Figure 3, satisfactory agreement is obtained between the theoretical and experimental responses, and Table 2 details the apparent rate constants as a function of electrolyte concentration and scan rate.

**Table 2:** Dependence of the Apparent Standard Heterogeneous Electron Transfer Rate Constants, *k*_app_^o′^, and Peak-to-Peak Separation on Scan Rate and Sr(NO_3_)_2_ Concentration

	*E*_pa_ – *E*_Pc_ (mV)		*k*_app_^o′^ (s^–1^)	
scan rate (mV s^–1^)	10 mM Sr(NO_3_)_2_	1000 mM Sr(NO_3_)_2_	10 mM Sr(NO_3_)_2_	1000 mM Sr(NO_3_)_2_
1000	93 ± 12	60 ± 10	10.9 ± 1.1	19.7 ± 2.1
500	84 ± 10	40 ± 10	6.4 ± 0.7	17.2 ± 2.0
100	50 ± 10	10 ± 10	2.5 ± 0.3	12.5 ± 1.3

For both electrolyte concentrations, the apparent rate constant is larger for higher scan rates. This behavior arises because ferrocenes located closer to the electrode surface, which have a higher rate of heterogeneous electron transfer, dominate the CV response at shorter timescales (faster scan rates). Significantly, for the more concentrated electrolyte solution, *k*_app_^o′^ is between approximately 2- and 5-fold higher for all scan rates investigated. Given that neither the availability nor movement of electrolyte ions appears to control the redox switching rate, this result suggests that the average electron transfer distance is shorter for the higher electrolyte concentration. This behavior could arise due to more efficient screening of the negative charge on the DNA backbone by the higher electrolyte concentration allowing it to approach the electrode surface more closely. However, cyclic voltammetry is limited since the DNA conformation could change as the applied potential is scanned if the time constants for conformational change and the experiment are comparable. Additionally, it is challenging to extract detailed kinetic information, especially with respect to the effect of the initial potential, and hence DNA conformation, on the apparent rate constant.

### Interfacial Capacitance

One of the key issues to understanding the role of electrostatics is to determine the charge on the electrode surface. At the potential of zero charge, PZC, there is no excess charge at the interface, while it will be negatively or positively charged at applied potentials that are more negative or positive than the PZC, respectively. It is known that the adsorption of a monolayer at the electrode/solution interface alters the double-layer structure and consequently changes the double-layer capacitance, *C*_dl_. The potential dependence of *C*_dl_ can be used to determine the potential of zero charge. Here, the capacitance was measured using small-amplitude potential step chronoamperometry. In these experiments, the potential was stepped from an initial value, *E*_i_, with a pulse amplitude of 25 mV, and the current response was recorded from microseconds to longer timescales. Successive measurements were performed, increasing the *E*_i_ value monotonically by 25 mV from 0.000 to 0.700 V. The pulse amplitude is sufficiently small to allow the measured capacitance to be regarded as an approximate differential capacitance. For potentials far from the ferrocene *E*^o′^, the step does not cause any change in the redox composition of the film, double-layer charging dominates the response and the current decays according to a single-exponential decay. For potential steps close to the ferrocene *E*^o′^, there are two time-resolved decays that correspond to the double-layer charging and the faradic reaction. The relatively slow rate of heterogeneous electron transfer, coupled with a short electrode response time, allows these processes to be resolved and fitting the early time data yields information about the resistance, *R*, and capacitance, *C*_dl_.

**Figure 4:**
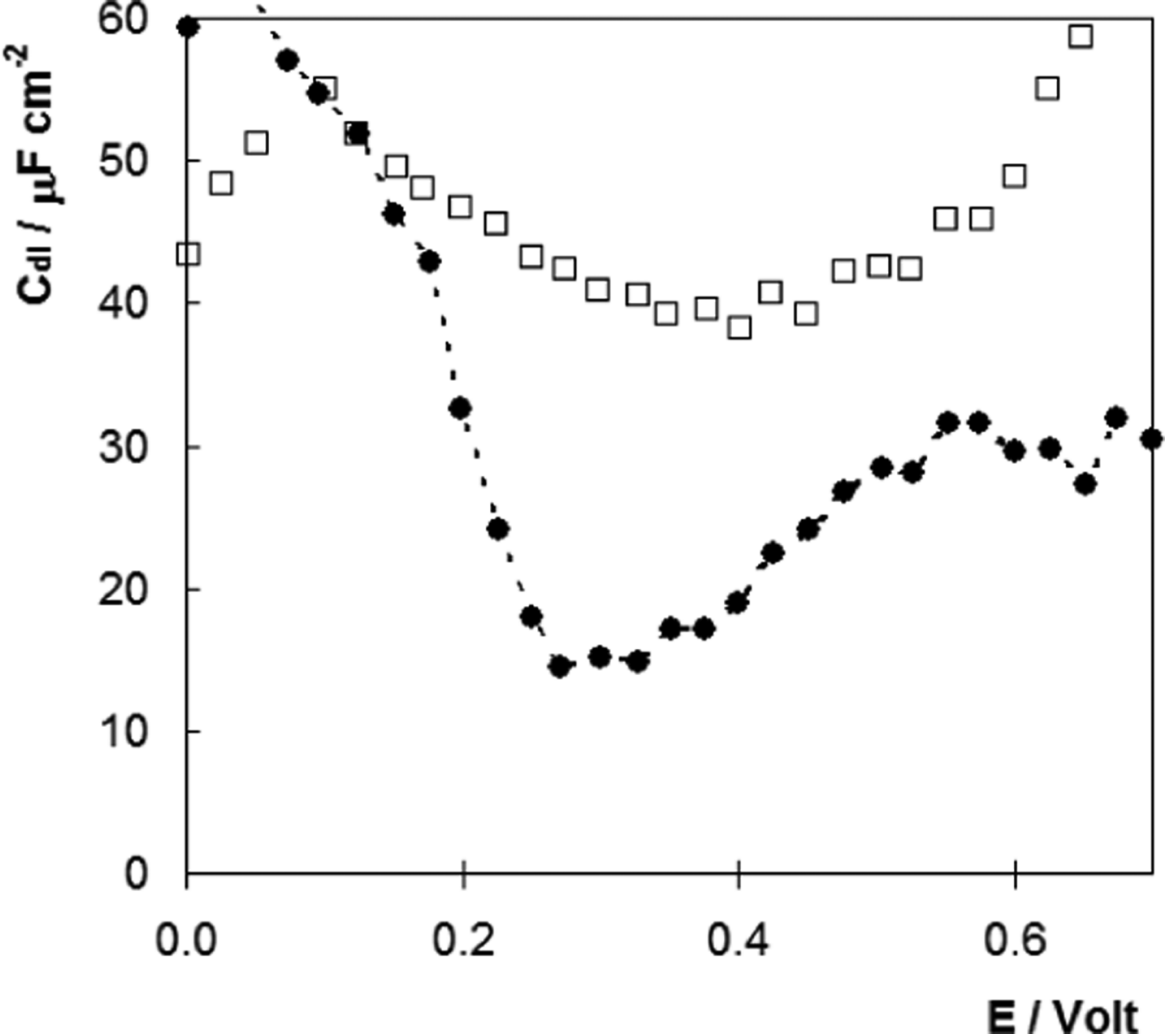
Dependence of double-layer capacitance, *C*_dl_, on the applied potential. Data for a pristine gold electrode are shown by □, and following modification with a DNA-Fc duplex by ●. The electrolyte is 10 mM Sr(NO_3_)_2_ (pH 6.5). The values are reproducible to within ±2 μF cm^–2^.

The resistance and the double-layer capacitance at each potential were determined using eq 1, where Δ*E* is the pulse amplitude, 25 mV.
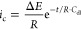
1Figure 4 shows the potential dependence of *C*_dl_ for both the pristine gold electrode and following modification with the DNA-ferrocene monolayer. The double-layer capacitance is lower for the DNA-modified electrode, which is consistent with the formation of a layer with a dielectric constant lower than water being formed at the interface, as well as ion displacement.^40^ Significantly, for both the modified and pristine electrodes, a local minimum in the *C*_dl_ is observed, which is better defined for the DNA-coated electrode. This minimum represents the PZC, where there is no excess charge on the electrode. DNA layer formation induces a shift on PZC, from approximately 415 to 275 mV. This behavior is consistent with the formation of a thiolated monolayer with hydroxyl groups (i.e., mercaptohexanol)^41^ and the adsorption of negatively charged molecules (i.e., DNA) at the electrode–solution interface.^42^

Significantly, the formal potentials of the ferrocene/ferrocenium couple, 321 ± 1 and 288 ± 7 mV, for 10 or 1000 mM Sr(NO_3_)_2_, respectively, are similar to the PZC, which means that the electrode is negatively charged when the monolayer is in the reduced, ferrocene, state and positively charged when the film is oxidized to ferrocenium. Thus, one might expect electrostatic repulsion of the ferrocenium sites by the positive electrode. However, the DNA backbone is negatively charged and its electrostatic interaction with the electrode will depend on the electrolyte concentration since a high electrolyte concentration will tend to screen the charge.

### Potential Step Chronoamperometry

The effect of the electrode–film electrostatic interactions, as controlled by the initial potential and electrolyte concentration, on the electron transfer dynamics was probed using chronoamperometry. For an ideal electrochemical reaction involving a surface-tethered species at a single distance, the faradic current following a potential step that changes the redox composition exhibits a single-exponential decay in time. For the ferrocene-modified DNA monolayers considered here, a more complex response is anticipated because the redox centers are located at different distances from the electrode surface.

**Figure 5:**
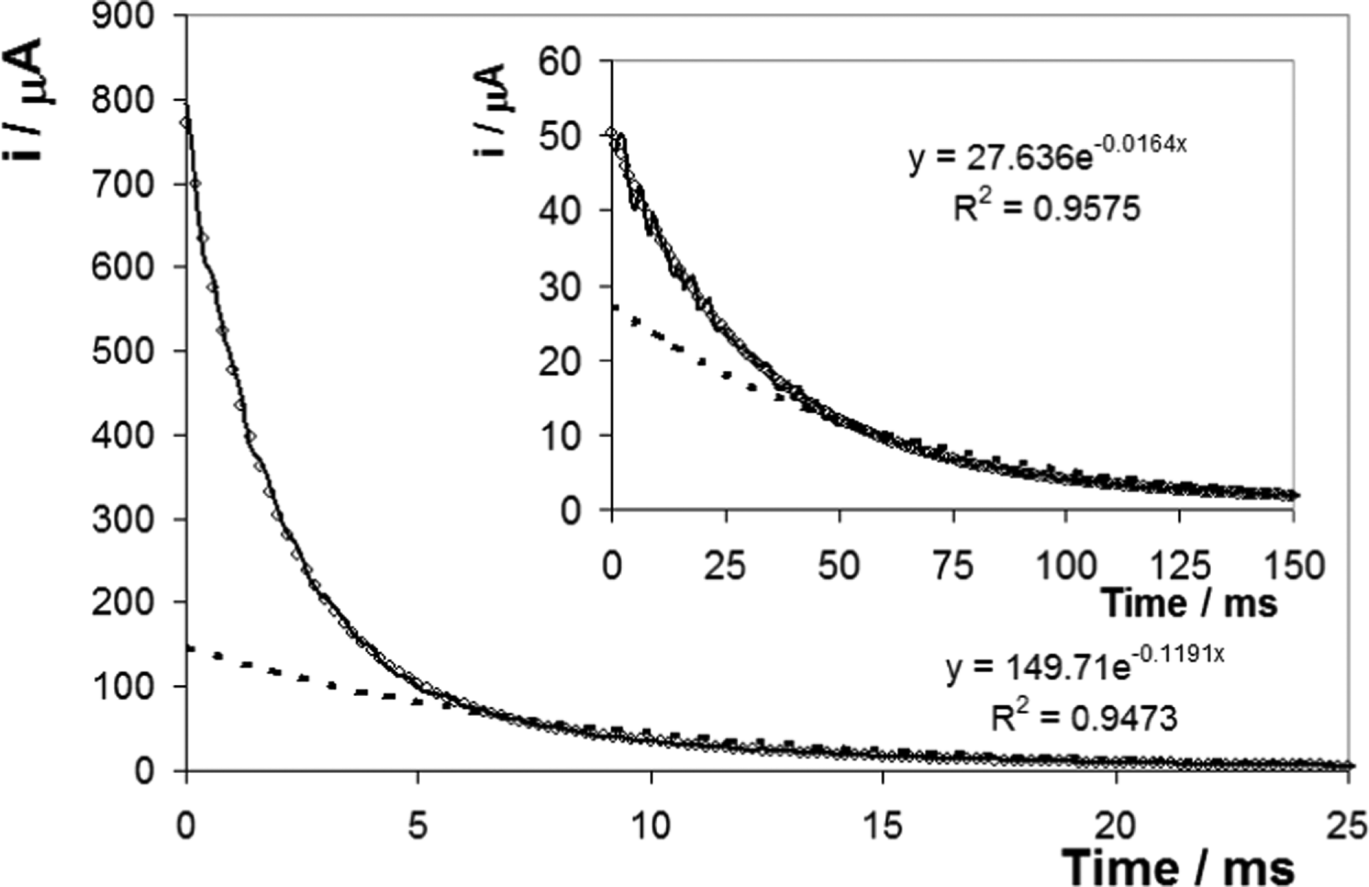
Current–time transients for the ferrocene-containing DNA monolayer where the supporting electrolyte is 10 mM Sr(NO_3_)_2_ (pH 6.5). The main figure shows the response where the overpotential is −0.046 V (step triggers ferrocenium reduction, fast process), while the inset is where the overpotential is +0.050 V (step triggers ferrocene oxidation, slow process). The solid line represents the experimental response, while the dashed line and open circles represent the best fit to single- and triple-exponential decays, respectively.

Figure 5 illustrates the effect of changing the initial potential on the chronoamperometry transients observed for the reduction of the ferrocene centers (main figure) (Fc^+^ + e^–^ → Fc) and the oxidation (inset) of the ferrocenium centers (Fc – e^–^ → Fc^+^), where the supporting electrolyte is 10 mM Sr(NO_3_)_2_ (pH 6.5). The uncompensated resistance is less than 50 Ω, which, in conjunction with the capacitance data shown in Figure 5, gives cell response times (product of resistance and capacitance) between 30 and 300 μs, i.e., at least 150 times shorter than the time constants for heterogeneous electron transfer. The dashed line, which represents the best fit to a single-exponential decay, clearly fails to adequately model the experimental data, i.e., the experimental response deviates significantly from that expected for ideal surface-confined species located at a single distance from the electrode.^42^ Ohmic drop can distort chronoamperometry responses since the flow of faradic and charging currents through a solution generates a potential that acts to weaken the applied potential by an amount *iR*, where *i* is the total current. This Ohmic drop can lead to severe distortions of experimental responses resulting in inaccurate measurements of the heterogeneous electron transfer rate. As illustrated in Figure 5, the faradic currents that flow in these chronoamperometry experiments are typically in the microampere range. Given that the uncompensated resistance is less than 50 Ω, an Ohmic drop of less than 5 mV is expected, which has a negligible impact on the current–time transients and does not explain the nonideal behavior observed.

For a surface-confined species at a single distance from the electrode surface, the current–time response is expected to follow single-exponential decay kinetics. This expectation is reflected in the fact that the transients cannot be satisfactorily fitted using a semi-infinite linear or even a mixed linear/radial diffusion model. Therefore, while recognizing its limitations, we have fitted a linear additive model using the minimum number of single-exponential decays required to obtain a satisfactory fit.

The response can be accurately modeled using a triple-exponential decay
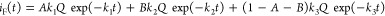
2where *A* and *B* are the fractions of the total charge passed, *Q*, for each of the electron transfer processes characterized by the three first-order rate constants, *k*_1_, *k*_2_, and *k*_3_. The fact that three components are sufficient most likely reflects the strong distance dependence of electron transfer, i.e., ferrocene centers located farther from the electrode surface do not contribute to the current response at these relatively short timescales.

In fitting the experimental responses, the fraction of each species and its associated rate are freely adjustable parameters (open circles in Figure 5). The observation that more than one rate constant is required is not surprising given that there are ferrocene centers along the duplex at different electron transfer distances, leading to different heterogeneous electron transfer rate constants. The individual rate constants and population fractions are given in Table 3.

**Table 3:** Dependence of the Rate Constants Extracted from Chronoamperometry Transients by Fitting a Triple-Exponential Decay on the Overpotential on the Concentration of Sr(NO_3_)_2_ as a Supporting Electrolyte^a^

electrolyte (mM)	initial potential (V)	overpotential (V)	*k*_1_ (s^–1^)	*k*_2_ (s^–1^)	*k*_3_ (s^–1^)
10	0.500	–0.050	580 ± 42 (0.50)	191 ± 14 (0.30)	80 ± 7.2 (0.20)
10	0.150	0.046	35 ± 2.3 (0.50)	13 ± 0.9 (0.30)	2.4 ± 0.2 (0.20)
1000	0.150	–0.052	25 ± 3 (0.65)	7 ± 0.5 (0.20)	2 ± 0.1 (0.15)
1000	0.500	0.056	604 ± 55 (0.60)	208 ± 18 (0.24)	90 ± 8.6(0.16)

a The fractional populations of each decay component are in parentheses.

**Figure 6:**
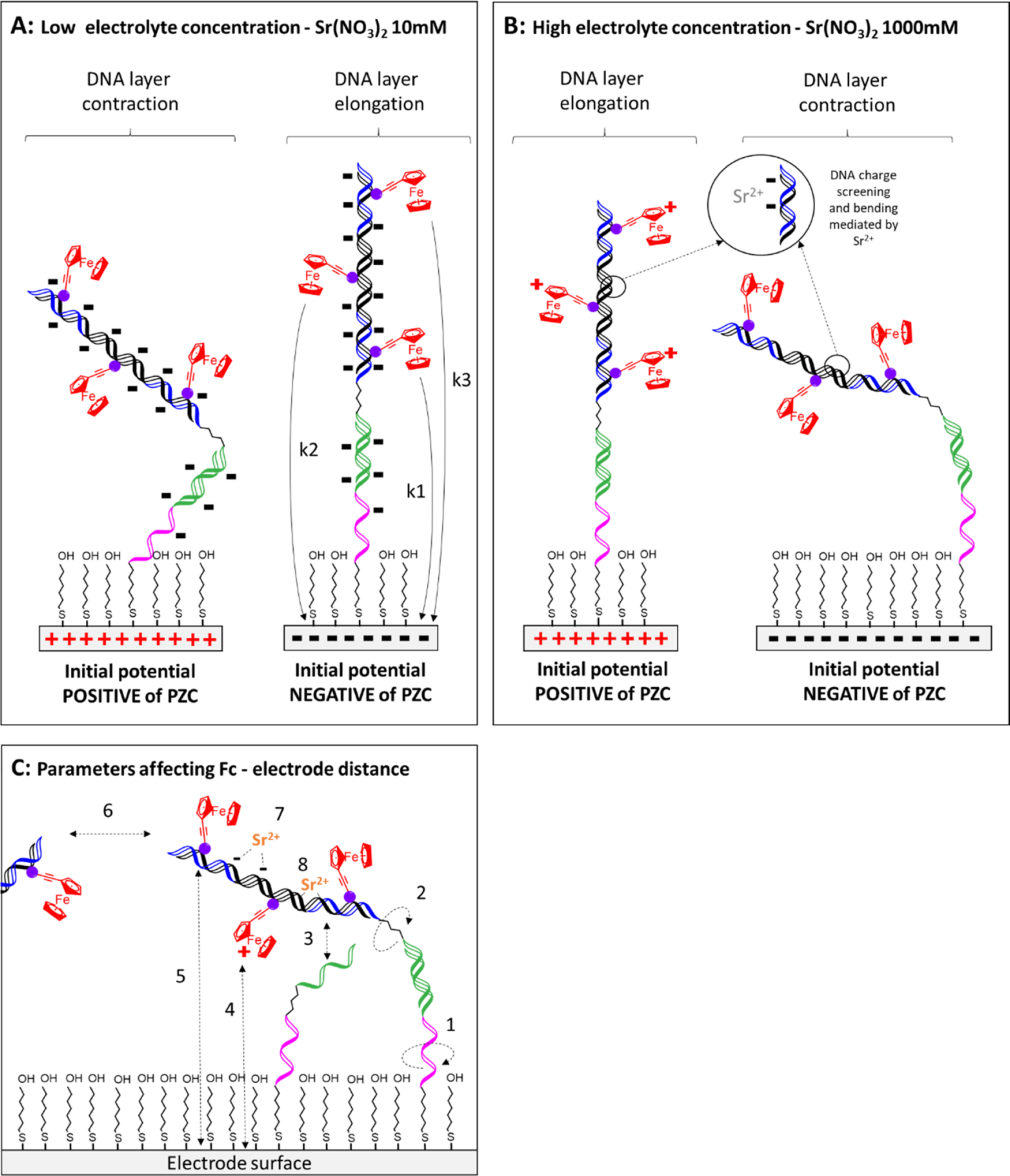
(A, B) Schematic representation of DNA layers when electrode surface has an initial potential positive or negative of the potential of zero charge, for low (10 mM) or high (1000 mM) electrolyte concentration. (C) Parameters affecting the distance between ferrocene tethered to the DNA and the electrode surface include high mobility of the poly T and alkyl linkers (1, 2), electrostatic repulsive or attractive forces between the ferrocene-labeled DNA strand, and ferrocene/ferrocenium centers with the capture probe layer (3), the electrode surface (4, 5), the neighboring dsDNA (6), the DNA phosphate backbone charge screening (7), and dsDNA bending (8) mediated by Sr^2+^.

The most striking feature of Figure 5 is that despite the absolute value of the overpotentials being indistinguishable in the two experiments, the rates of electron transfer depend on whether the monolayer is being reduced (highest rate 580 ± 42 s^–1^) or oxidized (highest rate 35 ± 2.3 s^–1^). Significantly, as shown in Figure 4, the PZC for the monolayer, 275 mV, is very similar to the formal potential of the ferrocene couple, 321 ± 1 and 288 ± 7 mV for 10 and 1000 mM Sr(NO_3_)_2_, respectively. Figure 6 shows the hypothetical initial DNA monolayer structure as a function of the initial potential and the electrolyte concentration before applying the potential step. Thus, prior to the potential step triggering oxidation of the ferrocene, Fc, to ferrocenium, Fc^+^, the electrode is negatively charged. When the concentration of the supporting electrolyte is low (10 mM) (Figure 6A), significant charge repulsion between the negatively charged DNA backbone and the negative electrode could cause the DNA to adopt an extended configuration, increasing the average electron transfer distances and giving rise to smaller rates of electron transfer. In contrast, for the reduction step, the electrode is initially poised positive of the PZC causing the negatively charged DNA monolayer to compress or concertina, bringing the ferrocenium centers closer to the electrode surface, leading to a higher rate of heterogeneous electron transfer.

**Figure 7:**
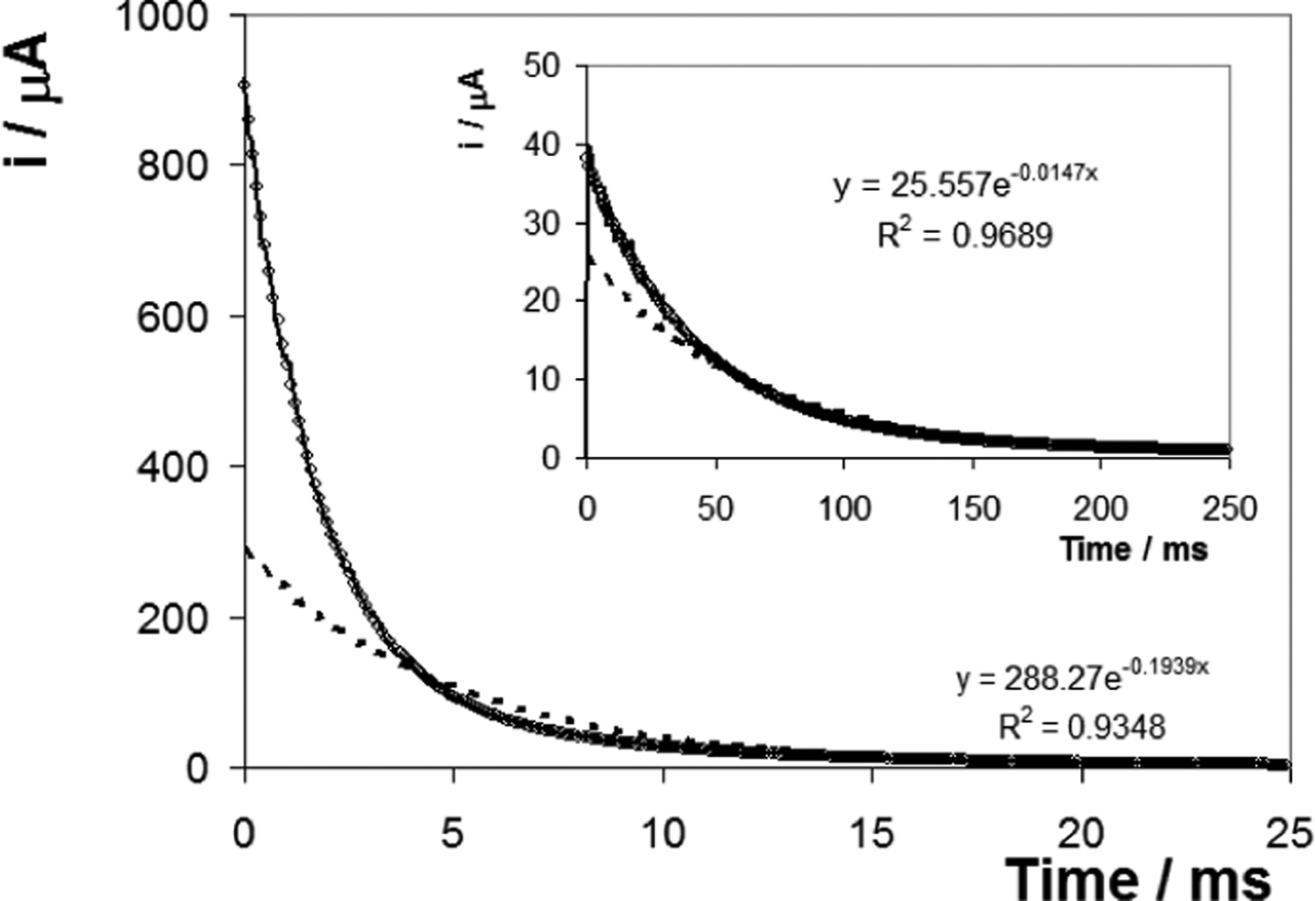
Current–time transients for the ferrocene-containing DNA monolayer where the supporting electrolyte is 1000 mM Sr(NO_3_)_2_ (pH 6.5). The main figure shows the response where the overpotential is +0.056 V (step triggers ferrocene oxidation, fast process), while the inset shows the response where the overpotential is −0.052 V (step triggers ferrocenium reduction, slow process). The solid line represents the experimental response, while the dashed line and open circles represent the best fit to single- and triple-exponential decays, respectively.

Figure 7 shows transients observed under similar conditions to Figure 5, except that the concentration of Sr(NO_3_)_2_ has been increased to 1000 mM. Increasing the electrolyte concentration does not significantly change the general features of the responses observed and the responses are well modeled by a triple-exponential decay. However, the effect of the initial potential is significantly different. Specifically, in contrast to the 10 mM electrolyte data, the rate of heterogeneous electron transfer for the reduction is lower than that observed for oxidation. This behavior could arise because the high Sr^2+^ concentration neutralizes or screens the charge present on the DNA backbone, making the DNA secondary structure more sensitive to the charge on the redox centers. At high electrolyte concentrations (Figure 6B), when the initial potential is positive of *E*^o′^, the positively charged ferrocenium centers are electrostatically repelled by the positively charged electrode and the rate of heterogeneous electron transfer for reduction is low. In contrast, for the oxidation reaction, the ferrocene centers are uncharged and can approach the electrode surface more closely, giving a faster rate of heterogeneous electron transfer.

### Potential Dependence of *k*

**Figure 8:**
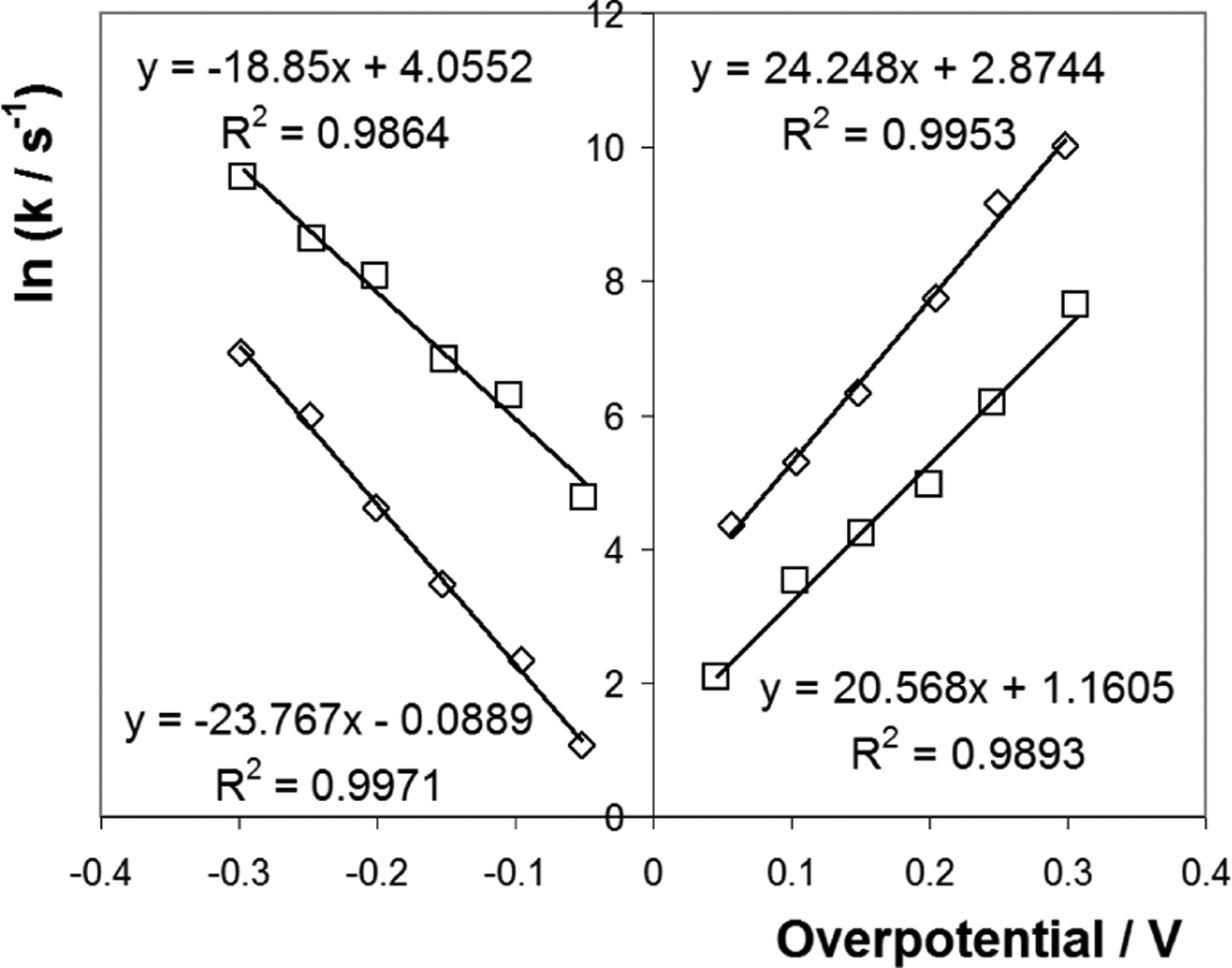
Dependence of the highest apparent rate constant on the overpotential. The supporting electrolyte is 10 mM Sr(NO_3_)_2_ (□) or 1000 mM Sr(NO_3_)_2_ (◇). Error bars are smaller than, or comparable to, the size of the symbols.

One of the great advantages of using chronoamperometry is that the redox composition of the ferrocene centers can be changed abruptly. This means that the response is not influenced by prior potential-induced changes, e.g., changes in the electron transfer distance as a function of the applied potential, which can occur when the potential is scanned in CV. Moreover, the driving force for electron transfer can be systematically varied by changing the overpotential. Figure 8 illustrates the Tafel plots of ln *k* vs overpotential, η, where the supporting electrolyte is either 10 or 1000 mM Sr(NO_3_)_2_ at pH 6.5. As discussed above, the decay of the current over time following a step that changes the redox state of the ferrocene centers follows a multiexponential model. For simplicity, only the fastest rate constant is considered here. Figure 8 shows that ln *k* depends approximately linearly on |η| for values up to approximately 300 mV. This behavior is consistent with the Butler–Volmer formulation of electrode kinetics with the slopes being equal to −α_c_*nF*/*RT* and (1 – α_a_)*nF*/*RT*, for the reduction and oxidation processes, where α_c_ and α_a_ are the cathodic and anodic transfer coefficients, respectively. In 10 mM electrolyte, the Tafel slopes yield α_c_ and α_a_ values of 0.48 ± 0.06 and 0.47 ± 0.05, respectively. In 1000 mM electrolyte, while the transfer coefficients sum to unity, the α_c_ (0.61 ± 0.06) and α_a_ (0.38 ± 0.05) values are statistically different from those expected for an ideal reversible reaction, 0.5, suggesting that the barrier to electron transfer is not symmetrical.

As illustrated in Figure 8, specifying the overpotential with respect to the formal potential determined using cyclic voltammetry gives rate constants that are not equal for zero overpotential. Figure 8 shows that in 10 mM Sr(NO_3_)_2_ electrolyte, for |η| = 0, apparent “standard” heterogeneous electron transfer rate constants of 246.3 ± 22.5 and 14.8 ± 1.1 s^–1^ were obtained for reduction and oxidation of the redox centers within the monolayer, respectively. In the 1 M electrolyte, the corresponding values are 7.8 ± 0.7 and 150.8 ± 11.6 s^–1^. The difference in apparent standard rate constants reflects the differences in the structure (electron transfer distance) that depends on the initial potential applied and the electrolyte concentration since these parameters control the electrostatic interactions of the DNA and the electrode.

The extent of DNA compression/expansion can be estimated using the apparent *k*^o^ values and a distance-dependent tunneling parameter, β, of 0.2 Å^–1^. Significantly, in 10 mM Sr^2+^, the DNA compresses (concertina closing) by approximately 50% on going from potentials that are negative of the PZC to positive values. In contrast, in 1000 mM Sr^2+^, this potential change causes the DNA to expand by approximately 60% (concertina opening).

### Effect of DNA Coverage

It is important to probe the effect of DNA surface coverage on the apparent electron transfer rates since interstrand interactions could significantly influence the behavior observed. Here, the DNA coverage was reduced even more by keeping the same capture probe surface coverage but diluting the deposition of the DNA-Fc solution to 0.2 μM while keeping the deposition time the same. Based on the charge passed in square-wave voltammetry, the surface coverage of the DNA-ferrocene is reduced by approximately 60%. Irrespective of the electrolyte concentration, the rate constants obtained at high and low DNA coverages are indistinguishable. While the significant electrostatic charge on the DNA is likely to keep them well separated as they are immobilized, it is important to acknowledge that the lower coverage may reflect the formation of DNA islands in which the local surface coverage is independent of the total number of molecules of DNA immobilized.^43,44^ However, the results obtained are consistent with lateral interactions between adjacent DNA strands not being very significant in controlling the rate of heterogeneous electron transfer.

## Conclusions

DNA monolayers labeled with redox centers represent an attractive system for understanding the influence of electrostatic interactions between an underlying electrode and the DNA. Here, we demonstrate that the rate of electron transfer between remote ferrocene groups attached to DNA and the electrode surface is strongly influenced by the electrostatic interaction between the charges DNA/redox centers and the electrode. When the electrode potential is poised negative of the potential of zero charge and the negative charge of the DNA is not screened (low electrolyte concentration, 10 mM Sr^2+^), the rate of electron transfer is low, possibly reflecting DNA in an extended state. Stepping to a potential positive of the PZC triggers compression of the DNA monolayer due to electrostatic attraction between the positive electrode and the negatively charged DNA. In sharp contrast, a high concentration (1000 mM) of Sr^2+^ effectively screens the charge on the DNA backbone. Thus, when the potential is positive of the PZC, electrostatic repulsion of the oxidized ferrocenium centers by the positively charged electrode causes the DNA to adopt an extended configuration. Stepping the potential to a negative value (wrt the PZC) causes the DNA to compress. Thus, the DNA-ferrocenium assembly acts as an interesting electrically and chemically controlled AND gate in which the four inputs (potential positive and negative of PZC as well as high and low electrolyte concentrations) dictate the rate of electron transfer. The rate of electron transfer can reach 10^4^ s^–1^ for large driving forces in the compressed DNA state. Typically, the rate of electron transfer in the “on” (compressed) and “off” (expanded) states differs by a factor of 16–20-fold under identical driving forces. In summary, we demonstrated that the electrolyte composition, concentration, and initial potential are key parameters to control the DNA layer at the electrode surface to enable electron transfer.
